# Challenges in HIV-1 Latent Reservoir and Target Cell Quantification in CAR-T Cell and Other Lentiviral Gene Modifying HIV Cure Strategies

**DOI:** 10.3390/v15051126

**Published:** 2023-05-09

**Authors:** Amanda M. Buck, Tyler-Marie Deveau, Timothy J. Henrich, Amelia N. Deitchman

**Affiliations:** 1 Division of Experimental Medicine, University of California San Francisco, San Francisco, CA 94110, USA; 2 Department of Clinical Pharmacy, University of California San Francisco, San Francisco, CA 94110, USA

**Keywords:** CAR-T cells, HIV-1 cure, HIV-1 envelope expression, lentiviral vectors, gene modification, eradication, immunotherapy

## Abstract

Gene-modification therapies are at the forefront of HIV-1 cure strategies. Chimeric antigen receptor (CAR)-T cells pose a potential approach to target infected cells during antiretroviral therapy or following analytical treatment interruption (ATI). However, there are technical challenges in the quantification of HIV-1-infected and CAR-T cells in the setting of lentiviral CAR gene delivery and also in the identification of cells expressing target antigens. First, there is a lack of validated techniques to identify and characterize cells expressing the hypervariable HIV gp120 in both ART-suppressed and viremic individuals. Second, close sequence homology between lentiviral-based CAR-T gene modification vectors and conserved regions of HIV-1 creates quantification challenges of HIV-1 and lentiviral vector levels. Consideration needs to be taken into standardizing HIV-1 DNA/RNA assays in the setting of CAR-T cell and other lentiviral vector-based therapies to avoid these confounding interactions. Lastly, with the introduction of HIV-1 resistance genes in CAR-T cells, there is a need for assays with single-cell resolution to determine the competence of the gene inserts to prevent CAR-T cells from becoming infected in vivo. As novel therapies continue to arise in the HIV-1 cure field, resolving these challenges in CAR-T-cell therapy will be crucial.

## 1. Introduction

Combination antiretroviral therapy (ART) has significantly reduced HIV-1 morbidity and mortality. However, latent viral reservoirs persist, composed largely of cells that do not express significant levels of viral antigens, thereby evading immune-mediated eradication [1]. These viral reservoirs persist indefinitely through a variety of homeostatic or other proliferative mechanisms in virally suppressed individuals, contributing to low level inflammation associated with numerous long-term comorbidities. In light of these ongoing comorbidities in the setting of ART and the massive global burden of HIV-1, developing curative therapeutic approaches remains a high research priority.

Gene modification therapies are at the forefront of HIV-1 cure strategies. While there has been success in CCR5-delta-32 mutation autologous stem cell transplants (SCT) in curing a handful of individuals [2,3,4,5], this strategy is practical only for those who require allogeneic stem cell transplantation for hematologic illnesses and have HIV-1 that exclusively uses CCR5 for cell entry [6]. To reduce residual HIV-1 burden and achieve long-term ART-free viral remission, various gene modification therapies have promise. For example, gene modification of stem cells during autologous stem cell transplant that disrupt one or more stages of the HIV-1 life cycle are currently being implemented. In addition, chimeric antigen receptor (CAR) T-cell therapy, which involves lentiviral vector delivery to autologous T cells, is a major HIV-1 cure strategy of interest due to the success of this approach for various hematologic malignancies [7]. This approach involves genetically engineering patients’ T-cells to express CARs on the cell surface which can recognize and bind to specific proteins expressed on HIV-infected cells leading to potential cell-mediated toxicity and immune-mediated clearance of infected cells [8,9,10]. 

CAR-T-cell strategies have potential advantages for boosting immune system response to HIV-1. This includes their ability to recognize cell surface proteins, given that they are based on heavy and light chain regions of the neutralizing antibodies or surface CD4 protein [11,12,13]. CAR-T cells may also be engineered to target other macromolecules apart from stably expressed cell surface proteins [14]. It is not entirely clear if classically designed CAR-Ts can recognize other antigens expressed with intracellular processing and major histocompatibility complex (MHC) presentation. As a result, novel strategies are being developed that target MHC-peptide complexes and do not compete with endogenous TCR for CD3 complex formation [15,16,17,18,19]. However, HIV-1 regulatory protein expression can lead to the downregulation of MHC 1, a process which occurs in more actively infected cells [20,21,22,23,24]. Nonetheless, prior research has demonstrated the potential for CAR-T cells to be able to both traffic to the diverse areas of tissue that compose the latent reservoir (e.g., lymph node, gut-associated lymphoid tissues) and play a vital role in long-term viral surveillance [25,26,27,28,29,30,31,32,33]. 

The potential for CAR-T-cell therapy to target HIV-1-infected cells through HIV-1 envelope gp120 regions in vivo comes with numerous technical challenges. HIV-1 envelope protein gp120 sequences are highly variable given antibody-mediated immune pressure [34,35,36], and thus, require CARs that recognize and engage a wide variety of envelope variants. The conserved region of gp120 is within this protein trimer and is not exposed until CD4 binding and identifying the target on latently infected cells that may differ across tissues [37,38,39]. While less of an issue for original CAR-T cells that used CD4 molecules to bind to HIV-1 envelope, CARs based on neutralization antibodies are likely to have variable binding to various viral strains. Another challenge is the identification and quantification of target cells when using lentiviral gene delivery to create CAR-T cells. This issue arises due to the sequence homology between HIV-1 and lentiviral vectors used to generate CAR-T cells [40]. This is an issue in lentiviral vector CAR-T cells as well as other non-CAR-T lentiviral gene modification strategies directed toward HIV-1 cure. 

In this review, we aim to systematically describe the current challenges facing therapeutic development of CAR-T cells for potential use as a curative therapeutic [41] with an emphasis on the challenges of targeting gp120 and vector-HIV-1 sequence homology on the development and implementation of quantitative assays.

## 2. Materials and Methods

This systematic literature review was performed according to the Preferred Reporting Items for Systematic reviews and Meta-Analyses (PRISMA) guidelines. PubMed literature library was searched for articles pertaining to gene modification therapies in the context of HIV-1 infection. 

The first search was conducted for background on the topic of gene modification and CAR-T cells. PubMed was searched for [“HIV-1”, “gene modification”, “therapies”, “CAR T-cell”]. The second search conducted was for the technical challenges associated with quantifying target cells. PubMed was searched for [“HIV-1”, “gp120”, “cell-surface”, “targeting”], [“HIV-1”, “sequence homology”, “lentivirus”], and [“HIV-1”, “lentivirus”, “CAR T-cell”, “resistance genes”]. The final search conducted was for gp120 targeting approaches and difficulties. Pub-Med was searched for [“gp120 conserved” “gp120 variable” “HIV bNAbs” “gp120 presentation” “HLA presentation” “CD4 inhibition” “gp120 conformation”.

## 3. Results

Due to the heterogeneous nature of the HIV-1 latent reservoir and scarcity of infected cells in people with HIV (PWH) on ART, there are many challenges with immune targeting of viral persistence. This review will address three different technical difficulties associated with quantifying and characterizing HIV-1-infected or lentiviral vector-transduced T cells. 

### 3.1. Challenges of Targeting HIV-1 gp120 for Infected Cell Clearance

HIV-1 is a retrovirus with the capacity to integrate into the host chromosomes. Current treatments such as ART allow PWH to suppress a large majority of circulating plasma HIV-1 RNA and reduce replication in tissues [42,43,44]. Rebound viremia occurs rapidly, however, following ART discontinuation in most people [45,46]. Despite ART-mediated viral suppression, ongoing immune dysfunction and inflammation persist [47,48,49,50,51,52,53,54]. Identifying and targeting the persistent reservoir is tantamount to long-term ART-free HIV remission. While HIV-1-infected cells can express high levels of gp120 during active replication [55], these levels are far lower in the setting of ART suppression. Expression of HIV env proteins on a cell’s surface may occur during initial virus-cell binding and entry, viral assembly, and budding, or to a likely much lesser extent, intracellular antigen processing and HLA-mediated presentation [56,57]. However, immunoPET imaging has recently demonstrated that low levels of HIV-1 or SIV gp120 protein can be identified across a range of tissues in the setting of ART using gp120-specific antibodies [58,59,60,61]; whether or not this reflects cell surface expression of gp120 or free viral proteins or virions in close anatomical proximity to residual infected cells is not known. The potential paucity of gp120 expression on cell surfaces makes CAR-T-mediated immune targeting difficult in people on suppressive ART. Given the hypervariability of large portions of HIV-1 gp120 due to its surface location on the virion and humoral immune mediated pressure, this glycoprotein is difficult to target across PWH, even when using a HIV-specific broadly neutralizing antibodies (bNAbs).

#### Challenges in Identifying and Quantifying HIV-1 gp120 in the Setting of ART

Most PWH have increased gp120 expression after ART cessation as virus begins to emerge and replicate in blood and tissues [58,61]. Ultimately, HIV-1 expression that is recognizable to the human immune system remains vital in the immune targeting HIV-1 cure strategies such as CAR-T approaches.

Gp120 is a large trimeric envelope protein expressed on the surface of the HIV-1 virion and infected cells, with three glycoprotein subunits including V1, V2, and V3. These variable loop regions protect HIV from immune recognition and assist in HIV virion binding for invasion into CD4 cells [37,62]. During virion binding to CD4, portions of this trimeric protein are released, and expose a conserved region of the envelope. This region is protected from recognition due to its sequence and functional consistency. Due to the exposure of these epitopes only in the late stages of cell infection, it is difficult to target this region for viral integration prevention and early neutralization. While immunotherapies have been brought into the HIV-1 cure field, the high level of immune escape has hindered the success of targeting these infected cells. Original CAR-T cells designed to recognize HIV envelope utilized CD4 proteins, but more recent CAR-T constructs have evolved to have broader and longer lasting antiviral activity by presenting costimulatory molecules (i.e., 4-1BB and CD28) combined with bNAbs as seen in Table 1 [41,63,64]. Another application of bNAbs is through leveraging single chain variable fragments (scFv) in CAR-T constructs. Increased success has been seen in persistence and protection in vivo of combination scFv CAR-T cells versus monotherapy. These scFv CAR-T cells allow for the recognition of conformationally available HIV-1 gp120 activated in an MHC-independent manner [65]. The affinity and specificity of the groups are also dependent on the positioning of the various domains as determined by the linker. This positioning is crucial in developing effective CAR-T therapies while preventing off-target effects or toxicities [66]. In addition, these variable domains fall victim to the same shortcomings that their parent monoclonal and bNAbs faced, an inability to accurately target the gp120 protein. A current study is using lentiviral vectors to mediate CAR-modification of T cells to present these anti-HIV CARs (i.e., duoCARs) and make cells resistant to HIV-1 [41,67]. Other novel CAR-T-cell technologies are being developed to recognize, bind, and kill MHC–antigen complexes, but these methods are predominantly in the pre-clinical development stage for various malignancies [15,16,17,18,19].

**Figure 1:**
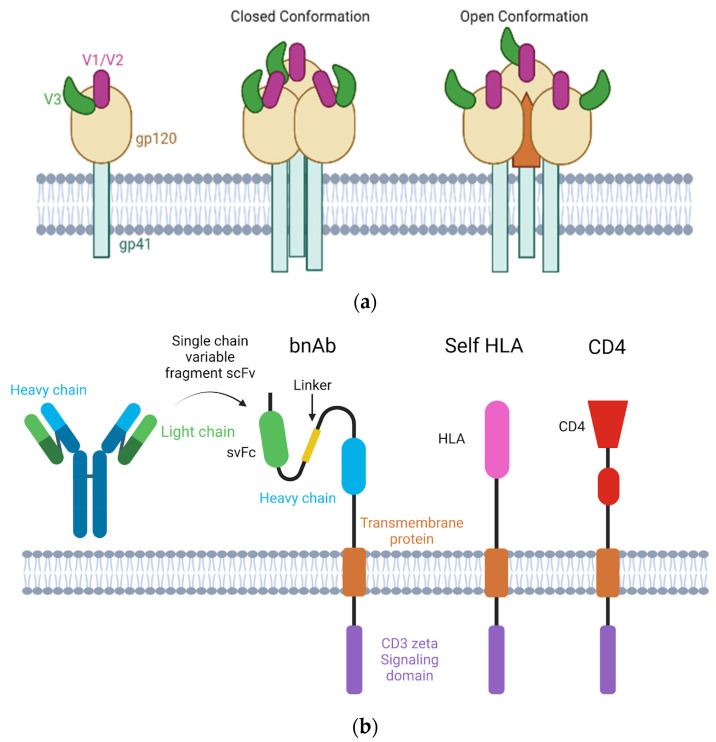
Structure of HIV envelope protein and TCR structures for CAR-T cells. (**a**) (**Left**) structure of HIV-1 envelope gp120 and gp140 proteins alone, (**middle**) full trimeric structure of the closed conformation before CD4 binding, and (**right**) trimeric protein in the open conformation exposing the internal epitope after CD4 binding (for simplicity, the release of gp120 and gp140 is excluded in this diagram). (**b**) Depiction of the composition of the CAR-T-cell receptor using the light and heavy chain of the antibody, as well as the transmembrane and intracellular proteins. The proceeding receptors show the hypothesized self HLA-presenting receptor model, then, the CD4-presenting model. Figure was created with BioRender.com (accessed on 28 March 2023).

There is a paucity of data regarding the frequency and density of gp120 expression on latently infected cells. In addition, quantifying the density of gp120 expression presents a distinct challenge as there is a major dearth of literature demonstrating surface detection of HIV-1 gp120 in the setting of ART outside of whole body immunoPET studies [58,68]. Without understanding its expression or density on persistently infected cells or the dynamics of viral envelope expression following treatment cessation, therapeutic development of CAR-T cells will be hampered. This also holds true outside of PET imaging. The inability to target gp120 consistently and efficiently, especially in latent tissue reservoirs, creates a barrier for virus and infection clearance [69]. As previously mentioned, the monoclonal antibodies and bNAbs developed thus far have difficulty targeting the full range of envelope subunit diversity [1,70,71]. The conserved regions of the HIV envelope are hidden within the tripod-like structure of gp120 and is only visible when actively bound and infecting a CD4 cell, as depicted in Figure 1. The lack of ability to use standardized antibodies to target the major region for HIV identification greatly reduces the ability to both identify and quantify expression infected human cells as well as for in vivo immune targeting. Similarly, bNAbs sometimes fail to target a conserved region across all infected cells in the human population [72,73,74], even if expressed in the setting of robust latency reversal or analytical treatment interruption. As CAR-T-cell therapeutics are commonly composed of variable antibody heavy and light chain regions, they are likely to have similar targeting issues. However, as a “living drug”, they have the capacity to expand in the presence of antigen recognition and may have different tissue penetration patterns to bnAbs or native T cells (e.g., central nervous system, lymph nodes, bone [58]). As a result, CAR-T-cell therapies (and bnAbs) are commonly designed to be used in combination with analytical treatment interruptions (ATI; i.e. highly monitored antiviral pauses) in which gp120 expression may increase dramatically [75].

### 3.2. Sequence Homology Challenges

Another technical challenge associated with CAR-T-cell development is using vector backbones for the integration into the host genome. While there are other ways to genetically engineer CAR-T cells, lentiviral vectors are used for the transduction efficiency and accuracy of multi-gene integration. In cancer therapy, the genus and family of lentiviruses do not create difficulty in quantifying and assessing therapeutic results; however, this is not the case for HIV-1 cure therapies. Here, we detail an overview of lentiviruses and the challenges their sequence similarities bring to therapeutic action. 

#### 3.2.1. Retroviruses and Their Conserved Regions 

The family Retroviridae is composed mainly of simple (gamma-retroviruses) and complex (lentiviruses) retroviruses [76]. The main difference between simple and complex retroviruses is the amount of polyproteins encoded that affect viral synthesis (e.g., viral RNA replication) [76,77,78]. Ubiquitous polyproteins encoded for in all retroviruses are gag, pol, env genes; while lentiviruses also are comprised of various other proteins, depending on the specific virus and strain, and include tat, tax, rev, rex, nef, etc., [76,77,78]. Both subfamilies contain gag, which maintains viral structure, pol, which encodes enzymatic ssRNA, and env, which encodes viral envelope proteins (gp120) [79]. These three regions are quite homologous across retroviral genomes. 

Common retroviruses used as vector backbones today are HIV-1 and murine leukemia virus (MLV) [80,81,82,83,84]. In this review, we will focus on the lentivirus subgroup, which includes HIV, due to the multiple advantages of lentiviral vectors that tend to be lacking in gamma-retroviral vectors. 

**Figure 2:**
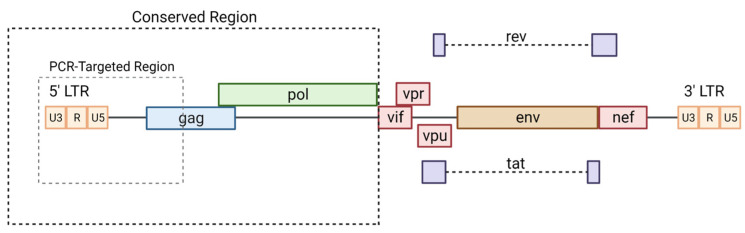
Conserved regions of HIV-1. The conserved regions of HIV-1 are targeted in PCR assays as well as commonly used in lentiviral vector production. Modified from [87] and created with BioRender.com (accessed on 1 May 2023).

In addition to proximal HIV-1 Gag [85], a conserved region that is shared by many lentiviruses is that of the long-terminal repeat (LTR) region that acts as a promoter and modulator of viral transcription [86]. These conserved regions of lentiviruses are shown in Figure 2. As depicted, the conserved regions tend to be consistent across the genus with little variation.

These conserved sequences are the core of the lentiviral backbone. This framework proves to be useful for a few reasons. One of them being the ability to carry and integrate large amounts of transgenes into the human genome. The size of the lentiviral vector backbone tends to be around 3.8 kb without any gene inserts. And while with increasing size of genes added tend to decrease transduction efficiency, each vector can hold around 9 kb in gene inserts, totaling approximately 14 kb [88,89]. This is more than the packaging capacity of typical adeno-associated viral vectors (4.8 kb of added DNA) [90]. More gene combination possibilities may allow a broader range of potential therapeutic effects. 

Another useful ability of lentiviral vectors is that they have long-term transgene expression and consistent transduction in replicating and non-replicating cells which is a core reason they are widely used in the field of gene-engineering [91,92,93]. In the past, viral vectors used on target cells that have had inconsistent expression of genes caused off-target effects on non-targeted tissues [94,95,96,97]. However, there are more updated lentiviral vector backbones that allow for the regulation of specific gene expression, such as the tet system [98]. As lentiviral vectors continue to be researched, integrated genes have sustained expression allowing for greater therapeutic effect in gene-modification therapies [99,100].

Due to the advances and advantages of lentiviral vector backbone usage, their use is widespread in developing CAR-T-cell therapies, including the HIV-1 cure field. These vectors are currently being used in human studies (ClinicalTrials.gov #NCT02797470, #NCT04648046, #NCT02343666, #NCT00569985, #NCT05529342). For instance, AIDS Malignancy Consortium 097 study (AMC097) modifies autologous stem cells of HIV-1 infected individuals with a lentiviral vector backbone that carries a CCR5 shRNA, chimeric macaque-human Trim5α, and a HIV-1 TAR decoy [101,102,103]. However, with the many lentiviral vector backbones being created from HIV-1, this creates downstream sequence homology issues with HIV-1. 

#### 3.2.2. Co-Quantification of HIV and Lentiviral Vector in HIV-1 Infected Individuals 

In the setting of lentiviral vector or HIV-1 quantification in samples from participants by various sequence specific PCR methods in gene therapy studies, the conserved regions of both viral sequences are often used, as hypervariable regions are not reliable enough to ensure accurate counts across participants [104]. For the quantification of HIV-1, the LTR-proximal Gag region is typically used for consistency and to reliably quantitate target copy numbers across the diverse viral strains (of note, lentiviral vector DNA and RNA is conserved, although infecting HIV-1 strains are highly diverse). Since primers and probes in PCR techniques are usually designed for conserved sequences, the vast majority of highly tested assays to define the HIV-1 reservoir correspond to these conserved regions common to lentiviral vectors [85].

To be able to quantify lentiviral vector and HIV-1 infection separately, there is a challenge to design assays that will not have cross-reactivity between conserved regions of HIV-1 and lentiviral vector sequences. If there is overlap in these regions, current assays are not able to differentiate between cell-associated lentiviral vector DNA and integrated HIV-1. Possibilities such as adding viral genes in the backbone or targeting smaller regions within the vector could lead to less cross-reactivity. It is also possible for viral load assays that detect lentiviral RNAs from the proximal Gag region to detect certain lentiviral vector transcripts. Not knowing the difference between the HIV reservoir and the presence of a lentiviral vector can cause a multitude of issues that affect the overall knowledge of therapeutic efficacy.

There are both clinical and research assays for quantification of HIV-1 that depend on conserved regions of HIV-1 sequence. While there are antibody, antigen, and nucleic acid tests in clinical use to detect and quantify virus in plasma, the HIV-1 reservoir is typically detected via RNA and DNA PCR of peripheral mononuclear blood cells (PBMCs) [105,106,107,108,109,110]. In PWH on suppressive ART, blood plasma virus is typically undetectable by routine viral load testing [111,112,113]. Cell-associated HIV infection can nearly always be detected, however. With the sequence homology of HIV-1 and various lentiviral constructs there is no current standard assay to distinguish between the two, prohibiting accurate monitoring of HIV-1 reservoirs following lentiviral-based CAR-T-cell therapy. This applies to the recently described HIV-1 Intact Proviral DNA Assay (IDPA) [114]. Furthermore, the inability to distinguish certain regions of HIV-1 from various lentiviral vectors presents challenges in characterizing the expansion of the dynamics of CAR-T-cell populations within the body following infusion with or without ATI. 

When conducting studies or clinical trials for gene-modification therapies, it is important to have a method to understand how these cells are expanding and exerting therapeutic and off-target effects on the body. There is an urgent need for standardization of quantitation assays that will be used for CAR-T-cell therapies for the current and future HIV-1 cure investigations. 

#### 3.2.3. Lentiviral Vector Uses in HIV Cure 

The field of gene modification therapies is a rapidly expanding field across diseases, and the HIV-1 cure space is currently adapting many of these techniques [115,116,117,118]. While there are a multitude of ways to genetically modify cells, the exploration of lentiviral vectors is being employed toward HIV cure. As previously discussed, use of lentiviral vector backbones in these therapies has several advantages. These uses include ease in which they can integrate large transgenes and facilitate expression within dividing target cell types [79,119]. However, in the clinical space, they are also ideal due to the thoroughly conducted safety and efficacy studies in humans [120]. 

Lentiviral-based gene modification is used to generate CAR-T cells for HIV-1 cure in patients who are otherwise healthy and stably suppressed on ART (ClinicalTrials.gov #NCT04648046) [41,67]. While the cells transduced with lentiviral vectors make RNA transcripts that code for CARs or other products of interest in gene therapy studies, they are engineered to avoid lentiviral replication [79,121]. Nonetheless, there is a possibility that gene modified CD4+ T cells become infected with HIV-1 in vivo. Although the potential for recombination between HIV-1 and lentiviral vector DNA is highly improbable due to advances in lentiviral vector production, the potential is there, which could theoretically pose safety and efficacy issues in clinical spaces [92,122,123,124].

### 3.3. Challenges of Single-Cell Resolution of CAR-T Cell Resistance Genes

Quantifying CAR-T-cell expansion and the correlative HIV-1 burden is critical to determining therapeutic success. Several major questions remain unanswered, however. For example, despite strategies to engineer CAR-T cells to be resistant to HIV infection (CAR-T cells are often CD4-T cells of origin) by using C46 or non-CD4-based single chain variable fragments (scFv) [41,67,125], it is not known if CAR-T cells or other gene modified cells (e.g., the multiple gene-modified stem cell transplant strategy as above) become infected in vivo, especially during potentially high levels of HIV-1 plasma viremia and replication in studies involving ATIs. 

#### Limitations of Current Single-Cell Analysis Methods 

Single-cell characterization of HIV-1-infected and CAR-T cells will be required in order to determine the dynamics of CAR-T cells (or other gene modified cell strategies that involve engineered CD4+ T cells or hematopoietic stem cells [101,102,103,126]) over time in vivo with precision and accuracy as well as to determine if these modified cells become infected in vivo. Whereas there are numerous methods to investigate host and viral transcriptional activity on a single cell level, there are limitations associated with them when looking specifically at lentiviral vector-mediated CAR-T cell and other cellular therapies in PWH. The main techniques that can distinguish attributes of individual cells are single-cell RNA sequencing, fluorescence microscopy, and flow cytometry [127,128,129,130]. 

Single-cell RNA sequencing surveys the sequence of individual cellular RNA transcript expression. scRNA seq has advantages in that it can identify distinct genetic and phenotypic cell populations. However, it is limited by cost because the relatively fewer number of cells that can be analyzed at once adds to the difficulty in detecting non-polyadenylated HIV-1 or lentiviral transcripts [131,132,133]. Fluorescence microscopy can detect the genetic profile and protein expression in culture and tissues samples, while flow cytometry utilizes antibody kinetics to analyze surface proteins [98]. An additional technique utilizing both scRNA seq and flow cytometry is cellular indexing of transcriptomes and epitopes (CITE) sequencing [99]. The application of these methodologies enables single-cell resolution for different research targets. CAR-T cells used in oncology research were previously used in methods to identify and track cells by using flow cytometry [67,100,134]. When there is optimal engineering of the CAR to have a uniquely identifiable biomarker (i.e., CD19 CAR-T cells), flow cytometry can quantify the changes in CAR-T-cell reservoir. An activated and discrete biomarker is also needed in other lentiviral gene modification strategies when there is no distinct surface protein that delineates modified cells. Being able to identify modified cells infected with HIV-1 is critically important as this indicates that modifications that are engineered to protect cells from HIV infection (e.g., C46 with CAR-T, multiple gene modification in AMC097) are not effectively preventing de novo infection. So while these assays provide useful information on single-cell dynamics and biology, there are limitations to each in the context of developing lentiviral-based CAR-T-cell therapeutics.

Assays that allow for high throughput quantification of HIV-infected cells (which can be as rare as one in a million CD4 T cells in peripheral blood) and gene-modified lentiviral vector at a single-cell level are highly desirable to advance the development of lentiviral CAR-T-cell therapies in the HIV field. These assays need not be sophisticated in terms of the multiomic capabilities, but rather allow the survey of a large number of cells for simultaneous characterization of HIV-1 and lentiviral vector insert DNA or RNA transcripts. Single-cell encapsulation in microfluidic droplets with subsequent multiplexed PCR detection of distinct target sequences that do not overlap is one potential, cost-effective solution.

## 4. Discussion

CAR-T-cell therapies are gaining traction in the HIV-1 cure field and hold some promise to target and remove infected cells expressing HIV-1 envelope proteins. While the safety, efficacy, and efficiency of lentiviral vector-generated CAR-T cells have been proven in other therapeutic areas, such as oncology, there are many practical assay issues that arise with the introduction of these modalities for HIV cure. HIV-1 envelope surface expression is likely low and metastable in the setting of suppressive ART and it is not clear if CAR-T cells can effectively target infected cells in vivo across a range of tissues. As a result, many studies involve ATI following CAR-T infusion to allow for easier recognition of infected cells and to maintain or expand the CAR-T cell pool. There are also many technical difficulties in measuring target protein expression (e.g., Env gp120) and to quantify lentiviral vector and HIV-1 DNA or RNAs both in bulk samples with single-cell resolution.

## Figures and Tables

**Table 1:** Generation of CAR-T cells [10]. Each panel describes the basic construction of the four generations of CAR-T cells and demonstrates their advancements.

First Generation	Second Generation	Third Generation	Fourth Generation
Ligand/scFv-based	scFv-based	scFv-based	scFv-based
One signaling domain	Two signaling domains	Three signaling domains	Three/four signaling domains
CD3ζ signaling domain	CD3ζ signaling domain	CD3ζ signaling domain	CD3ζ signaling domain
	CD28 or 4-1BB costimulatory domain	CD28 costimulatory domain	CD28 costimulatory domain
		4-1BB costimulatory domain	4-1BB costimulatory domain
			Other costimulatory domains or ligands

## Data Availability

No new data were created.
